# The Physician’s Highest and Only Calling Is to Heal the Sick

**Published:** 1897-05

**Authors:** J. H. Allen

**Affiliations:** Chicago, Ill.


### "THE PHYSICIAN'S HIGHEST AND ONLY CALLING IS TO HEAL THE SICK."

J. H. Allen, M. D., Chicago, Ill.

First, the call is to a physician, not to the minister, nor to the judge, the lawyer, or pedagogue, yet he must be all of these in one; he must have the powers and qualifications of them all; he must be able to minister unto their wants, in sickness, in sorrow, and in distress of body, mind, and soul. He must be a high court, sitting in its majesty of power, judging as to diet, to labor, or to rest, to hearing, thinking, seeing; to all the problems of life in its many phases and complexities, and multi-relationships of even life and of death; the probability of things present and things to come. He must hold a horoscope of our lives and our futures in his knowledge of the sciences of causes and of effects. He must be a lawyer in his reasonings, for he is frequently placed in the witness-box, and must plead his own case; questions that would puzzle a philosopher he must answer on the spur of the moment, with no warning, or perhaps no forethought; yet his answers must be plausible and scientific, for the patient frequently understands the phenomena and the consequences as well as he does, though they are at a loss to analyze it. So he reasons with them, pro and con, and at last succeeds in leaving them in a mist of technicalities and verbose terminolgies and unknown medical phrases; and lastly, what school teacher teaches in the short hour or half-hour of his visit so much knowledge, while all the time from out of a chaos of words he is constructing a cosmos, a diagnosis, a prognosis, a pathogenesis, a pathopoesis, symptoms grouped and symptoms classified, symptoms weighed and symptoms measured. The evidence objective, physically, mentally, or morally. The evidence subjective, with all its possibilities and probabilities; the relationship to husband, wife, child, or family; their relationship to God, the world, and the devil; to eating and to drinking, or to all their environments. He must follow up that slippery and uneven path of life that "has chattered over stony ways, in little sharps and trebles," working out the problems (the *prima causa*) of the disturbed life force to which all his ills are heir. Such is his calling, such is his mission, such is his duty, and he who falls short of fulfilling this noble trust, this highest mission among men, has not a true conception of his calling.

In the second place, he is called to "heal the sick," not to treat well people, nor to help well people out of their difficulties, into which they have drawn themselves; nor to cover up sins which they have committed with his professional cloak, but to help sick ones out of their difficulties and sins that they have committed, or rather the effects of sin (disease). He must always be a defender of the wronged and of the right, a knight-errant in his full armor, always optimistic, never pessimistic; pointing to the sunshine, never to the shadow; holding aloft ever the banner of hope, never of despair, and when hope is dead, turn their shallops toward the blessed isles "where dwells the Great Ulysses that we know." Called to heal the sick, not to poison their already vitiated and weakened system with crude drugs, or, in other words, chemicalize them; not to narcotize them, or opiate or anesthetize them into unconsciousness, for coma is not sleep, nor paresis of the central organs of the nervous system a cure for pain; for consciousness and cure are homeopathic, and unconsciousness and palliation antipathic. Neutralization is cure, palliation is paralyzation often. One is law in action, which is life; the other is license to benumb, to produce coma, to destroy consciousness of pain or suffering by the suicidal process of temporarily suspending life by the mechanical or chemical action of crude drugs; out of one process life is evolved, and out of the other death is evolved. The drug may be or is good, but the evil arises in not applying it through law to restore the disturbed life forces. Therefore good and evil are death, which brings us back to Adam's primary sin. A knowledge of good and evil proved to be death. On the other hand, we find in Homeopathy that the drug is good, and the law is good; therefore a knowledge of good only is life. Choose, then, will you, as honest men of science, that out of which is evolved life, or that out of which is evolved death. The great French surgeon who followed Napoleon all through his conquests, and whose name I have forgotten, was called personally by the Emperor as he was about to evacuate Egypt, to see six French soldiers who had all been mortally wounded in the abdomen by the explosion of a shell. They were beyond help, and could not be removed, and the Emperor, knowing this, said to the great doctor, give them something and end their suffering. The great physician looked at the Emperor as if he was about to obey his request, but suddenly his professional honor and pride began to arise in his breast, and he quietly said to Napoleon, "My mission is to save life, not to destroy it," and the baffled Emperor left them to their fate, and moved on to other duties, having in this little incident fully learned the true office of a true physician. So let us fully learn that fact, that we are called to save life, not to take life, nor to destroy it. For only He who gave life, only He alone in whom we live and breathe and have our being shall cancel it, shall cut the Gordian knot that dissolves its relationship with its physical edifice. All things were first thinkings. There are two kinds of thinkings, good thinkings and bad thinkings. Good thinking brings us enduring and lasting benefit, a longer life in the physical and the spiritual. But the fruits of bad thinking are pain, suffering, degeneration, and physical and spiritual death. Primary, then, all disease was first bad thinking, whether put into action or not. Some of these are venery, lust, avarice, gluttony, greed, disobedience, all coming from within the man by gratifying the desires of the mind and body, out of which has developed all the chronic miasms which are the primary bases of all disease in the human organism, either directly or indirectly, mentally, morally, or physically, mental aberration, functional disturbance, or structural change. If this be true, then a broad knowledge of these things is absolutely necessary in order to cure disease and not suppress it, to save life and not to hasten death.

